# Test‐Retest Reliability, Learning Effect and Measurement Error of the Modified Shuttle Test (MST) in Children With Asthma: A Multicenter Study

**DOI:** 10.1002/ppul.71852

**Published:** 2026-10-01

**Authors:** Lara Bezerra Radis, Vitória Cavalheiro Puzzi, Thaila Corsi Dias, Natielly Soares Correia, Jéssica Lane Felippe Montes, Mariana Mazzuca Reimberg, Fernanda Cordoba Lanza, Evelim Leal de Freitas Dantas Gomes, Cláudio Luiz Castro Gomes de Amorim, Karina Couto Furlanetto

**Affiliations:** ^1^ Associated Graduate Program (MSc and PhD) in Rehabilitation Sciences at State University of Londrina (UEL) and University Pitágoras UNOPAR (UNOPAR) Londrina Paraná Brazil; ^2^ University Nove de Julho, UNINOVE São Paulo São Paulo Brazil; ^3^ Department of Physical Therapy Graduate Program in Rehabilitation Sciences, University Federal de Minas Gerais (UFMG) Belo Horizonte Minas Gerais Brazil; ^4^ Graduate Program Science Rehabilitation Universidade de São Paulo São Paulo São Paulo Brazil; ^5^ Health Science Center, State University of Londrina (UEL) Londrina Paraná Brazil

**Keywords:** asthma, exercise tolerance, pediatrics, reliability

## Abstract

**Objective:**

To investigate test‐retest reliability, measurement error (standard error of measurement [SEM] and minimum detectable difference [MDD]), and the learning effect of the Modified Shuttle Test (MST) in children with asthma.

**Methods:**

Multicenter cross‐sectional study including clinically stable children aged 6–12 years with asthma. Lung function and exercise capacity were assessed using MST. Intraclass Correlation Coefficient (ICC) was calculated to assess test‐retest reliability. The first and second tests were compared using the paired Student's *t‐*test to assess differences between tests. SEM and MDD were calculated to quantify measurement error.

**Results:**

One hundred and twenty three children were included (71 boys [57%]; median age 9 [7–10] years). The mean distance covered in the best MST was 611 ± 214 meters (confidence interval [95% CI] = 573–649meters). The MST showed an excellent test‐retest reliability (ICC = 0.96; 95% CI: 0.95–0.97). Distance was significantly lower in the second test than in the first (574 ± 218 vs. 601 ± 216 m; *p* = 0.0001). Learning effect was −27 ± 79 (4% ± 14%), the SEM was 56 meters (9.5%), and the MDD was 156 m (26.6%).

**Conclusion:**

The MST demonstrated excellent test–retest reliability and acceptable measurement error for assessing exercise capacity in children with asthma. Although a systematic difference was observed between tests, there was no evidence of a consistent learning effect, with individual variability in repeated performance.

## 1 Introduction

Asthma is a heterogeneous chronic inflammatory disease, characterized by variable respiratory symptoms, including wheeze, shortness of breath, chest tightness, and cough, associated with variable expiratory airflow limitation. It is considered one of the main respiratory diseases of childhood, generally developing in the first years of life and imposing a substantial burden on children, families, and healthcare systems [1, 2].

According to the Global Initiative for Asthma (GINA), the diagnosis of asthma in school‐age children should be based on a history of characteristic respiratory symptoms together with objective evidence of variable expiratory airflow limitations, which can be assessed using spirometry and other recommended tests [1, 2]. Poorly controlled asthma may lead to recurrent exacerbations, impaired physical activity, reduced quality of life, school absenteeism, hospitalizations, and, in severe cases, death [1, 2].

Although the goal of asthma management is to enable children to participate fully in physical activities, some children may experience reduced exercise capacity and exercise‐related symptoms, especially when asthma is not adequately controlled [3]. This capacity is extremely important in the lives of individuals, because it reflects the efficiency of the cardiopulmonary system in distributing oxygen and musculoskeletal efficiency during physical exercise [4].

To assess oxygen consumption, cardiopulmonary exercise testing (CPET) is considered the gold standard [5]. However, when CPET is not available, field tests such as the Modified Shuttle Test (MST) can provide a practical and valid alternative for evaluating exercise capacity [6].

The MST is a test carried out in a 10 m course, delimited by two cones, at progressive speed. The subject must walk between the cones as indicated by a beep. At the end of the test, the distance traveled and the speed are obtained, allowing the calculation of the child's oxygen consumption [6].

This test is widely used in healthy adult populations and in those with chronic lung diseases, with the best result of two tests being considered [7]. Although the MST has demonstrated convergent validity for assessing maximal exercise capacity in children and adolescents with asthma [8], its test–retest reliability and measurement error have not yet been established in this population. Furthermore, it remains unclear whether repeated testing results in a learning effect and whether a second test is necessary to obtain a reliable assessment [9].

Reliability analysis is essential to determine whether MST provides consistent measurements when repeated under similar conditions and, therefore, to support its use in clinical practice and research. In addition, quantifying measurement error is important to determine the magnitude of change required to exceed the expected variability of the measurement and to distinguish a true change in performance from measurement error. Although the MST is commonly performed using the best result from repeated tests, it remains unclear whether a second test is necessary to account for a potential learning effect in this population. Therefore, the aim of the present study was to investigate the test–retest reliability, measurement error (standard error of measurement [SEM] and minimum detectable difference [MDD]), and learning effect of the MST in children with asthma.

## 2 Methods

### 2.1 Sample and Study Design

This is a multicenter cross‐sectional study carried out in three research centers in Brazil: one in Londrina (Paraná), at the Research Center of the Anhanguera‐Unopar University (center 1), and two other centers in São Paulo (São Paulo)—the Allergy Outpatient Clinic of the Pediatrics Department of the Federal University of São Paulo (UNIFESP) (center 2) and the Laboratory of Respiratory Functional Assessment (LARESP) of the Latin America Memorial Unit (center 3)—both linked to the Nove de Julho University (UNINOVE). Data collection at Center 1 was prospective, whereas Centers 2 and 3 provided previously collected data that were subsequently shared with Center 1 for the present analysis. The sample comprised more than the minimum number of participants recommended by Consensus for Selection of Health Measurement Instruments (COSMIN), which states that the minimum number of participants for this type of study is 50 individuals [10].

The children evaluated at center 1 were recruited from the Specialties Outpatient Clinic of the University Hospital of the State University of Londrina (HU‐UEL) and through dissemination of the research through social networks. Children who met the inclusion criteria for centers 2 and 3 were recruited from the Allergy and Clinical Immunology Outpatient Clinic at UNIFESP. For the previously collected data from Centers 2 and 3, eligibility was confirmed based on the available study records and the predefined inclusion criteria.

The inclusion criteria were, cumulatively: children aged between 6 and 12 years, inclusive (i.e., they were between 6 and 12 complete years of age at the time of assessment); clinical diagnosis of asthma of all severity levels according to GINA [11]; absence of other lung diseases; having performed two MSTs by the same evaluator with a minimum interval of 30 min and recovery time to return to the baseline state of all vital signs; and clinical stability (i.e., no exacerbations for at least 30 days, defined as not having used reliever (rescue) medication for acute symptom relief more than four times in the same day, no changes in controller medication during this period, and no hospitalizations due to asthma or other diseases).

The exclusion criteria were withdrawal of consent by the participant or legal guardian, or any physical, cognitive, or behavioral condition that prevented safe completion of the study assessments.

Data collection at center 1 was carried out between March 2023 and January 2024, while data from centers 2 and 3 had been collected between 2014 and 2019.

The data collection procedures at each center were approved by the respective institutional ethics committees (Center 1: opinion no. 5,754,939; Center 2: opinion no. 575,501; and Center 3: opinion no. 3,846,185). All participants provided informed assent, and their legal guardians provided informed consent at the time of data collection.

### 2.2 Evaluations

At the beginning of the evaluation, the following data were collected from all individuals: age, weight, height, sex, body mass index (BMI), Z‐Score from BMI, in addition to information provided by the legal guardians regarding demographic data, personal and surgical history, symptoms, use and dose of medication. The classification in relation to severity was performed by differentiating the Steps according to GINA [1]. The pulmonary evaluation was performed through spirometry according to international standards [12, 13] and the reference values for the pediatric population [14]. To verify dyspnea and fatigue during the test, the modified Borg Scale was applied [15].

### 2.3 Maximum Exercise Capacity Test

Maximum exercise capacity was assessed using the Modified Shuttle Test (MST). The test consists of walking or running back and forth along a 10 m course marked by two cones positioned at a 9 m distance, leaving half a meter on each side to account for the change of direction. Participants were instructed to walk around each cone and return toward the opposite cone, synchronizing their pace with standardized audio signals. The test comprises 15 incremental levels, with the speed progressively increasing as the interval between audio signals decreases. Consequently, participants may naturally progress from walking to running to maintain the required pace. The test was terminated when the child failed to reach the cone before the audio signal on two consecutive occasions, voluntarily stopped because of symptom limitation, or reached maximal exercise capacity. The procedure was also interrupted if oxygen desaturation or signs of severe exertion, such as cyanosis, pallor, or marked sweating, were observed [9].

Before and after the test, blood pressure, heart rate and peripheral oxygen saturation were monitored, and the child was asked about dyspnea and fatigue. The MST was performed twice by the same evaluator with an interval of at least 30 min and recovery time to return to the baseline value of all vital signs [4, 6]. This recovery period was adopted to minimize the influence of fatigue and allow participants to return to their baseline physiological condition before the second assessment. The outcome of the test was the distance covered, and the test with the best performance was used in the analyzes.

The maximum heart rate (HRmax) was estimated using the following formula for the pediatric population: 208 − (0.7 x age). Then, the percentage of HRmax was calculated considering the HRmax obtained during the MST. The estimated oxygen consumption (VO_2_) was calculated using the formula: VO_2_ peak(L/min) = −0.457+ (gender * 0.139) + (weight * 0.025) + (distance in MST * 0.002) [9].

Reference equations for the MST have been established for healthy children and adolescents, allowing exercise performance to be interpreted according to individual characteristics such as age, sex, and body mass index rather than absolute walking distance alone. The equation proposed by Lanza et al. [16]. has provided an important framework for the standardized assessment of functional exercise capacity in the pediatric population:

Predicted MST distance (m) = 845.559 + (193.265 × Sex) + (47.850 × Age) − (BMI + 26.179) [16], where sex is coded as 0 for girls and 1 for boys, age is expressed in years, and BMI in kg/m^2^.

### 2.4 Statistical Analysis

The distribution of data was analyzed using the *Shapiro–Wilk* test and the data were described according to their normality. For the reliability analysis, the Intraclass Correlation Coefficient (ICC) was calculated, with a 95% confidence interval (95%CI) and its upper and lower limits. The ICC values were interpreted according to the following cut‐off points: <0.50 = poor reliability; 0.50–0.75 = moderate reliability; 0.75–0.90 = good reliability; > 0.90 = excellent reliability [17]. The visual analysis was carried out using the Bland & Altman graph [18]. The paired Student's *t*‐test was used to compare performance between the first and second MST. The learning effect (LE) was calculated in absolute value (LE = [2nd test − 1st test] and in percentage (LE = [2nd test − 1st test]/1st test*100) [18].

The Standard Error of Measurement (SEM) was calculated with the formula: SEM = SD_difference_/√2 [19], where SDdifference is the difference between scores of the two measures.

The relative measurement error was expressed as: SEM% = SEM/X̄ * 100, where X̄ is the mean value of the two measures [20]. The classification adopted was: SEM < 5% = very good; from > 5% to <10% = good; from >10% to <20% = questionable; >20% = unreliable [19].

Furthermore, the Minimal Detectable Difference (MDD) was also calculated in absolute (MDD95) and relative values (MDD%) using the formulas: MDD = SEM × 1.96 × √2 [19] and MDD% = MDD/X̄ × 100 [20], where an MDD% <30% was considered “acceptable,” with values ≤ 10% considered “excellent.” The MDD95 represents the minimum change between two measurements required to exceed the expected measurement error at the individual level, with 95% confidence. A value of *p* < 0.05 was adopted as statistical significance. The study was reported in accordance with the STROBE Statement checklist for cross‐sectional studies, and the methodology followed the COSMIN guidelines [10].

## 3 Results

A total of 123 children diagnosed with asthma met the eligibility criteria and were included in the analysis, with 33 participants from center 1, 53 from center 2 and 37 from center 3. Participant selection is shown in Figure 1. The sample comprised 57% boys, with a median age of 9 years. Lung function characteristics are presented in Table 1.

**Figure 1:**
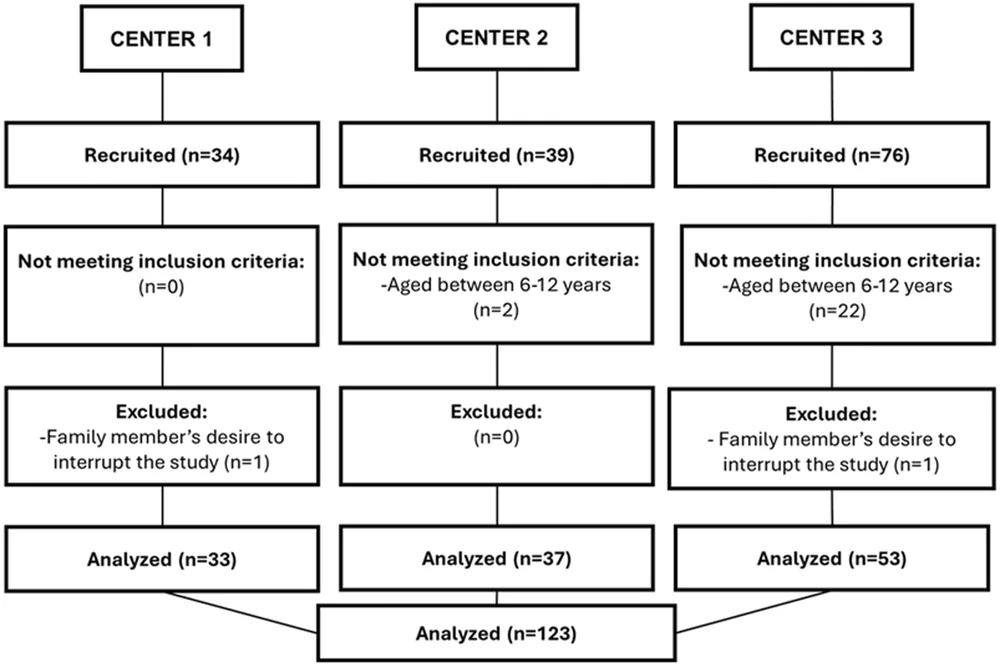
Flowchart of the study.

**Table 1:** Characteristics of the individuals included.

	*N* = 123
Anthropometric data	
Sex (male/female, *n*, %)	71 (57)/52 (43)
Age (years)	9 [7–10]
BMI (kg/meters^2^)	17 [15–21]
*Z*‐Score (from BMI)	0.77 [−0.01–1.98]
STEPS GINA^a^, *n* (%)	
I	17 (14)
II	31 (25)
III	43 (35)
IV	24 (20)
V	4 (3)
Lung function	
FEV_1_ (L)	1.75 [1.35–2.22]
FEV_1_ (%predicted)	95 [83–103]
FVC (L)	2.09 [1.71–2.55]
FVC (%predicted)	103 [90–112]
FEV_1_/FVC (%)	83 [77–88]

Abbreviations: BMI, Body mass index; Z‐score: FEV_1_, Forced expired volume in one second; FVC, Forced vital capacity.

^a^ Data were unavailable for four children.

MST performance in the first and second tests is presented in Table 2. The ICC for the MST was 0.96 (95%CI: 0.95–0.97). Learning effect and measurement error values, including the SEM and MDD are described in Table 3. The agreement and variability between the two tests, assessed using Bland–Altman analysis, are illustrated in Figure 2.

**Table 2:** Performance and reliability analysis of the Modified Shuttle Test.

Performance	1st test	2nd test	Best test
Modified shuttle test (meters)	601 ± 216	574 ± 218	611 ± 214
Modified shuttle test (predicted in meters)	1334 ± 149	1334 ± 149	1334 ± 149
Modified shuttle test (%predicted)	45 ± 15	42 ± 15	45 ± 14
Maximum HR percentage (%)	88 [78–94]	88 [76–95]	89 [78–95]
Borg final dyspnea (after)	3 [2–7]]	4 [1–6]	3 [2–7]
Borg final fatigue (after)	4 [2–7]	4 [2–6]	4 [2–7]
Estimated peak VO_2_ (L/min)	1.69 ± 0.58	1.64 ± 0.59	1.71 ± 0.57
VO_2_peak (predicted in L/min)	3.16 ± 0.55	3.16 ± 0.55	3.16 ± 0.55
VO_2_ peak (%predicted)	53.05 ± 13.45	51.20 ± 13.45	53.74 ± 13.22

Abbreviations: HR, Heart Rate; VO_2_, Oxygen Uptake.

**Table 3:** Metric properties of the modified shuttle test.

Metric properties	Values
ICC 95%CI (lower limit–upper limit)	0.96 (0.95–0.97)
Minimum detectable difference (meters)	156
Minimum detectable difference (%)	13
Standard error of measurement (meters)	56
Standard error of measurement (%)	5
Learning effect (meters)	−27 ± 79
Learning effect (%)	4 ± 14

Abbreviations: CI, Confidence Interval; ICC, Intraclass Correlation Coefficient.

**Figure 2:**
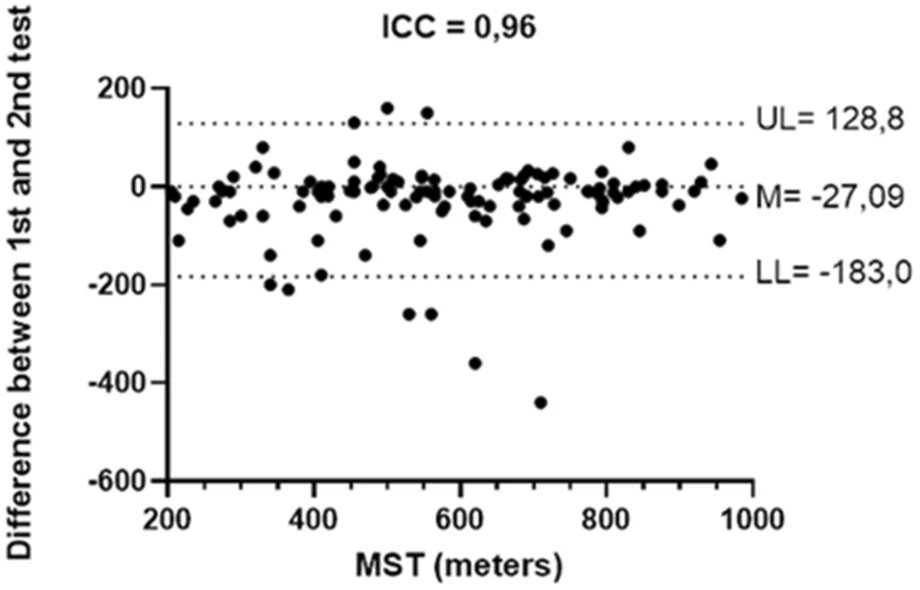
Bland & Altman intra‐rater visual analysis of MST. Legend: LL, lower limit; M, mean difference; MST, Modified Shuttle Test (mean); UL, upper limit.

The percentage of maximum HR was 84% in the first test and 85% in the second test. The mean distance covered was 601 ± 216 in the first test and 574 ± 218 in the second test, with a mean difference of −27 m (*p* = 0.0001). In addition, 65% of the participants achieved their greater distance in the first test, whereas 35% achieved their greater distance in the second test (Figure 3). Among the 43 children who performed better in the second test, the mean difference between tests was 27 ± 79 m.

**Figure 3:**
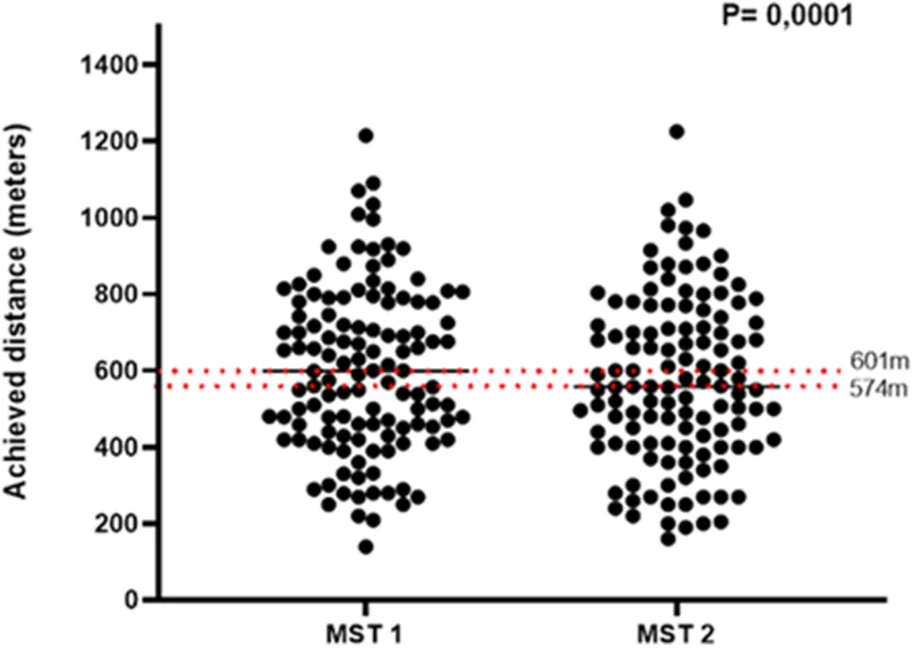
Comparison of distance performed between the 1st and 2nd Modified Shuttle Test. Legend: m, meters; MST, Modified Shuttle Test. MST 1 = 601 m; MST 2 = 574 m. [Color figure can be viewed at wileyonlinelibrary.com]

## 4 Discussion

This study investigated the test‐retest reliability, measurement error, and learning effect of the MST in children with asthma. The main findings were that the MST is a reliable field test for assessing exercise capacity in children with asthma and demonstrates acceptable measurement error. Although performance differed significantly between the first and second assessments, performance was lower on the second test, and no consistent learning effect was observed. This suggests that routine repetition of the MST solely to overcome a learning effect may not be necessary in this population. However, considerable individual variability in performance across repeated tests was observed.

The ICC results indicate that the test has excellent test‐retest reliability, suggesting that the MST provides consistent relative measurements when repeated under similar testing conditions. Importantly, the statistically significant difference between tests does not conflict with the high ICC, as the ICC assesses the relative consistency of participants’ rankings across repeated measurements, whereas the paired comparison evaluates systematic differences in absolute performance between tests. These findings extend the available evidence regarding the measurement properties of the MST in this population. In addition, the present study provides information on the magnitude of measurement error. The SEM was 56 m (9.5%), classified as good relative measurement precision according to the predefined criteria, while the MDD was 156 m (26.6%), which was within the acceptable range (< 30%). These findings provide complementary information to the ICC, because a high ICC alone does not indicate the magnitude of error associated with repeated measurements.

Regarding repeated performance, the distance covered was significantly lower in the second MST than in the first test, with a mean difference of 27 m which is substantially smaller than the minimum detectable difference of 156 m. The observed pattern does not correspond to the conventional learning effect, in which performance improves with repeated testing. Instead, the direction of the difference suggests a small systematic reduction in performance on the second assessment. Nevertheless, individual responses varied considerably, indicating that the effect of repeated testing was not uniform across participants.

Furthermore, to date, MST responsiveness and the minimum clinically important difference (MCID) have not been established in children with asthma. In this context, the MDD value obtained in the current study may help guide future clinical decisions. However, the MDD should be interpreted as an estimate of measurement error rather than as a threshold for clinically important change. Interestingly, the MCID of the MST has been investigated in children and adolescents with cystic fibrosis [21]. In that study, the authors proposed using the MDD_90_ (at least 97.08 m) as the criterion for interpreting a meaningful change in exercise performance, because this value was higher than the MCID (at least 60 m) established based on children's perception. Thus, a change exceeding the MDD_90_ would not only surpass the measurement error but would also exceed the magnitude considered clinically important by the children. Future studies should investigate MST responsiveness and establish the MCID in children with asthma.

We hypothesize that the lower performance on the second test may reflect factors unrelated to a learning effect, such as residual physical or psychological fatigue, despite the standardized recovery interval between tests. This behavior can be attributed to the child's age, which differs from adults who mostly show better performance on the second test [22]. However, these factors were not directly assessed in the present study and should therefore be interpreted cautiously.

Some individuals with asthma may experience reduced aerobic capacity and exercise intolerance, highlighting the importance of accurate assessment [23]. Although previous studies have established reference values, predictive equations, and convergent validity for the MST in children with asthma [9], evidence regarding other measurement properties remains limited. The present study addresses this gap by evaluating the test‐retest reliability, learning effect, measurement error, and repeated test performance in this population, providing additional evidence to support the clinical application of the MST.

The maximum HR percentage reached during the MST was similar between the two assessments (84% and 85%, respectively), supporting a comparable physiological and response across tests. In the present study, children's heart rate exceeded 80% in both tests, consistent with a maximal or near‐maximal exercise response. Although maximal exercise capacity is ideally assessed by CPET, which directly measures peak oxygen consumption (VO_2_ peak), field tests such as the MST provide a more accessible and feasible alternative for estimating exercise capacity when CPET is not available. Among field‐based tests for children and adolescents, the MST may be particularly useful because it allows estimation of VO_2_ through a validated equation while simultaneously providing an objective measure of functional performance, such as the distance achieved [8]. In the present study, the estimated VO_2_ obtained from the MST was approximately 50% of the predicted values based on age and body weight, suggesting reduced exercise capacity in the study population.

Results comparable to those of the present study have been reported in children with different chronic conditions, who also demonstrated reduced exercise capacity [24, 25]. The metric properties of the shuttle‐based tests have been investigated in in pediatric populations, including children and adolescents with cerebral palsy (CP) who use wheelchairs [25] and those classified in the Gross Motor Function Classification System (GMFCS) as level 3 [26]. In these cases, the 10‐Meter Shuttle Ride Test and the Shuttle Run Test were performed on the same day and proved to be reliable and valid [25, 26]. Similarly, the Shuttle Run test demonstrated good reproducibility in obese and non‐obese children aged between 8 and 10 [27].

Regarding test performance, children with cystic fibrosis showed no substantial learning effect between field test assessments performed on different days and, as in the present study, performed better during the first test [28]. A similar pattern was observed in obese children who performed two tests on the same day, with the first test resulting in better performance and a single test considered sufficient to assess maximal exercise capacity [27]. These findings are consistent with our results, in which most children covered a greater distance during the first assessment.

We consider the limitations of this study to be that MST reliability was assessed with the same evaluator and on the same day. Therefore, it was not possible to determine whether the results would remain consistent across different evaluators and/or days. Although vital signs were measured before the second test to ensure that they returned to resting levels, and a standardized interval of at least 30 min was established between the tests, residual physical or psychological fatigue cannot be completely excluded.

The strengths of the study include its multicenter design and relatively large sample for a pediatric asthma population. The findings provide clinically relevant evidence regarding the reproducibility and measurement error of an inexpensive and feasible field test for assessing exercise capacity in children with asthma.

The Modified Shuttle Test demonstrated excellent test–retest reliability and acceptable measurement error in children with asthma. Although a systematic difference was observed between the two assessments, there was no evidence of a consistent learning effect, with considerable individual variability in performance across repeated tests. These findings do not support the routine performance of a second Modified Shuttle Test solely to overcome a learning effect in this population.

## Author Contributions

**Lara Bezerra Radis:** conceptualization, investigation, funding acquisition, writing – original draft, methodology, validation, visualization, software, formal analysis, project administration, data curation, writing – review and editing, resources. **Vitória Cavalheiro Puzzi:** conceptualization, investigation, funding acquisition, writing – original draft, writing – review and editing, visualization, validation, methodology, project administration, formal analysis, software, data curation, supervision, resources. **Thaila Corsi Dias:** methodology, project administration, conceptualization. **Natielly Soares Correia:** conceptualization, methodology, software. **Jéssica Lane Felippe Montes:** conceptualization, methodology, data curation, writing – review and editing. **Mariana Mazzuca Reimberg:** supervision, resources, writing – review and editing. **Fernanda Cordoba Lanza:** writing – review and editing, supervision, resources, data curation, software, methodology. **Evelim Leal de Freitas Dantas Gomes:** writing – review and editing, supervision, resources, data curation, software, methodology. **Cláudio Luiz Castro Gomes de Amorim:** resources, supervision, writing – review and editing. **Karina Couto Furlanetto:** conceptualization, investigation, funding acquisition, writing – original draft, methodology, validation, visualization, writing – review and editing, software, formal analysis, project administration, data curation, supervision, resources.

## Funding

The authors have nothing to report.

## Ethics Statement

The collections were approved by the ethics committees of the three institutions involved (center 1 in Londrina (Paraná), at the Research Center of the Anhanguera‐Unopar University: opinion no. 5,754,939; center 2 in São Paulo (São Paulo)—the Allergy Outpatient Clinic of the Pediatrics Department of the Federal University of São Paulo (UNIFESP): opinion no. 575.501; and center 3 in Latin America Memorial Unit: opinion no. 3,846,185).

## Consent

All participants signed the Free and Informed Assent Form and their guardians signed the Free and Informed Consent Form.

## Conflicts of Interest

The authors declare no conflicts of interest.

## Data Availability

The data that support the findings of this study are available from the corresponding author upon reasonable request.
